# Compressive strength ratios of concretes containing pozzolans under elevated temperatures

**DOI:** 10.1016/j.heliyon.2024.e26932

**Published:** 2024-02-23

**Authors:** Ghasem Pachideh, Majid Gholhaki, Ahlam Aljenabi, Omid Rezaifar

**Affiliations:** a Sharif University of Technology, Iran; b Faculty of Civil Engineering, Semnan University, Iran; c Faculty of Civil Engineering, Semnan University, Iran

**Keywords:** Bentonite, Zeolite, Supplementary cementing materials, Temperature, Compressive strength

## Abstract

Cement production is one of the major pollution contributors owing to its large rates of energy consumption and gas emission. Moreover, high temperatures could detrimentally impact the concrete infrastructure and thus, it would be essential to study performance of such structures under exposure to the elevated temperatures. In this paper, post-heat performance of the concrete whose cement has been added by zeolite and bentonite at ratios of 6 and 10% (by cement weight) under exposure to temperatures of 28, 150, 300 and 700 °C, was studied. Based on the results, replacing cement by zeolite and bentonite at the age of 90 days under ambient temperature, increases the compressive strength compared to the control specimen. Moreover, it was observed that heating the cubic and cylindrical specimens containing 10% bentonite at 150 °C, increase the compressive strength by 40%. Conversely, the results indicate that when exposed to temperatures of 300 and 700 °C, a decreasing trend is seen in the tensile strength of both cubic and cylindrical specimens containing the pozzolans. Peak intensity of C–S–H has dropped as per rise in temperature from 28 to 700 °C. These values reveal that peak intensity of C–S–H up to 300 °C, is approximately the same but under 700 °C, it has reduced considerably. In all the cubic and cylindrical specimens, it can be seen that the specimens heated at 150° have the highest compressive strength and the specimens heated at 700 °C have the lowest compressive strength compared to the same unheated specimens. The XRD patterns at 150 and 300 °C, reveal decrease and increase in the Portlandite content the difference between conversion ratio of the cubic and cylindrical specimens in this study, to the values provided by the codes, is less than 10%.

## 1 Introduction

Concrete is one of the most widely used construction materials that is composed of cement, water and aggregates. In terms of both cost and environmental impact, concrete is a significant material, which is produced within a complicated process. Accordingly, cement is produced under a thermal operation (heated up to temperature of 1450 °C) and approximately, 710 kg CO_2_ is emitted as per production of each ton of cement [1]. To reduce the CO_2_ emission during concrete production, it would be vital to replace the cement with appropriate alternative materials such as cement-like materials. These materials contribute to the cement hydration and in this way, provide hardness for concrete. The mixtures with these characteristics are called supplementary cementing materials (SCM) among which, industrial by-products such as fly ash (FA) and silica fume (SF) are the most common ones [2].

Use of the pozzolanic materials in producing concrete is known as one of the optimal methods to mitigate emission of the greenhouse gases and avoid depletion of the energy resources as a result of which, cement consumption could be lowered. In this respect, pozzolans have received significant attention to be partially used as a replacement for aggregates in the concrete, by which the concrete properties could be enhanced as well [3]. Zeolite and bentonite are characterized as the natural pozzolans that are rich in AL_2_O_3_ and SIO_2_ which react with Ca (OH)_2_ and subsequently, create the C–S–H gel and aluminates. These materials are eco-friendly and their incorporation into the concrete mixture, results in a great rate of durability and mechanical performance [4]. In addition to being able to resist the seismic and wind loads, the structures are expected to withstand fire and its probable consequences. Fire could induce irreparable life and financial losses and thus, it would be of importance to have a proper insight towards concrete behavior under high temperatures.

In recent years, numerous researchers have attempted to investigate the effect of the pozzolans on mechanical properties of concrete. For instance, in 2019, Rahman et al. studied effect of the heated bentonite on mechanical properties of the concrete. In this study, bentonite was used as a replacement for cement at ratios of 0, 10, 15, 20, 25, 30 and 35%. Accordingly, the compressive and tensile strength tests were conducted on the specimens containing both heated and unheated bentonite. The results indicated that inclusion of the bentonite could desirably affect the concrete properties and it was found that use of thermal activation could increase mixing level of bentonite into the concrete. Additionally, it was perceived that in the case of specimens containing unheated bentonite, the optimal ratio of bentonite is 10–15% but in the case of the heated bentonite, this ratio increases to about 15–20% [5]. In 2020, Rezaifar et al. evaluated effect of partially replacing cement with bentonite and kaoline in the low-strength concretes. Based on the results, when content of bentonite and kaoline exceeds 2.7% (by cement weight), the tensile strength decreases by 75% compared to the control specimen [6]. Sheba et al. investigated into the effect of the fly ash and bentonite as the cement substitutes for mass construction of concrete. It was found that incorporation of 12.5% fly ash and bentonite could markedly result in heat generation while producing mass concrete such that by preparing this concrete mixture, compressive strength increased by 10% in comparison with 90-day strength of the control specimen [7].

In 2019, Trumer et al. studied the effect of a special type of bentonite on concrete performance under temperature of 900 °C and found that compressive strength of the hardened concrete with calcined clay, decreased at the early ages but to the contrary, at the age of 90 days, the strength of the bentonite-incorporated concrete increased [8]. Based on the results, as content of bentonite as the replacement for cement increases, in return, workability and water absorption of concrete are reduced [[9], [10], [11]].

Study of mechanical properties of a concrete specimen containing zeolite at ambient temperature revealed that a constant ascending or descending trend could not be expected [[12], [13], [14]]. The most important reason in this regard, concerns the differences in chemical composition of the zeolite, which is mainly dependent on location of exploiting the materials. In 2018, Barbara et al. concluded that the zeolite-incorporated concretes have a lower compressive strength compared to those excluding zeolite at the ages of 7 and 28 days. Nonetheless, rate of reduction at older ages of 90 or 180 days, is much less and in some cases is even equal to zero [15]. In 2019, Shahab et al. investigated the compressive strength of the concrete specimens containing zeolite and kaoline. They found that incorporation of 5–12% zeolite at all ages, decreased the compressive strength but, when 15% kaoline was added to the mixture, the strength started increasing. In general, it was concluded that the maximum compressive strength without using kaoline, is achieved when 7% zeolite is incorporated [16]. In 2020, Moghadam et al. evaluated the tensile and compressive strengths of concretes containing zeolite and SF under exposure temperatures of 28–800 °C. They used zeolite and SF at replacement ratios of 0, 10 and 20% and found that as the temperature elevates to 800 °C, the strengths are reduced and consequently, the water existing in specimens, starts evaporating. Promisingly, the results proved that replacing cement with zeolite and SF, in the concretes exposed to elevated temperatures, could lead to improvements in terms of both engineering and environmental issues [17]. In 2020, Kang et al. evaluated effect of zeolite with various replacement ratios on concrete performance. It was observed that inclusion of 10% zeolite with size of 5.6 μm at the age of 90 days, led to the best result in terms of compressive strength. Moreover, the zeolites with size of 5.6 μm resulted in better results on the compressive strength compared to the other sizes. Importantly, it was observed that incorporation of zeolite could reduce number of the contraction cracks [18]. In this respect, the compressive and bending strength of the specimens were evaluated after being exposed to high temperatures. The results indicated that addition of the granulated blast furnace slag (GBFS) to the mortar containing pumice increases the strength compared to the specimens excluding GBFS. Furthermore, at temperature of 900 °C, the compressive strength of mortars containing 80% GBFS decreased only by 23% whereas, in case that GBFS was not utilized, the compressive strength was lessened by 70% compared to the regular temperature [19].

In 2017, Gholampour and Ozbakkaloglu carried out a study on the strength and durability of the concrete containing large amounts of the FA (class F) and GBFS. In their study, the cement was replaced with the pozzolans up to 90%. The results indicated that as the amount of the FA increases from 50 to 90%, the compressive strength of concrete is significantly reduced. However, the compressive strength of the specimens containing 90% GBFS, is the same as the regular cases excluding pozzolans at the age of 28day. Moreover, they found that using 50 and 90% SF and GBFS, respectively, diminished the water absorption of the specimens [20].

Zeybek et al. [21] used the waste glass as a partial replacement for cement and different ratios (0%, 10%, 20%, 30%, 40%, and 50%) were tested in concrete production. The aim of their research was to investigate the effect of waste glass on the mechanical properties of concrete, including compressive strength, splitting tensile strength, and flexing strength. Workability and slump values were measured on fresh concrete. Cubic and cylindrical specimens were prepared and tested to obtain compressive strength and splitting tensile strength. Additionally, a three-point bending test was carried out on specimens to obtain the flexural strength. A 20% substitution of waste glass as cement showed the highest mechanical properties, while combined waste glass particles and crashed glass particles increased up to a certain level and then decreased due to reduced workability. They found that the optimum replacement level for combined waste glass was 10%. Meanwhile, they developed practical empirical equations to determine the compressive, splitting tensile, and flexural strengths of concrete with different amounts of waste glass.

To investigate the effect of waste ceramic powder (CP) on the flexural behavior of reinforced concrete beams (RCBs), Aksoylu et al. [22] produced twelve specimens with different amounts of mixing ratios. The longitudinal reinforcements percentage (0.77%, 1.21%, and 1.74%) and CP percentage (0%, 10%, 20%, and 30%) were chosen as parameters. CP could be effectively used up to 10% of cement as a replacement material. However, increasing the CP percentage by more than 10% reduced the load-carrying capacity, ductility, and stiffness of RCBs, especially when the longitudinal reinforcements percentage was high. At 0% CP, the load-carrying capacity reduced by 0.4%, while with CP increased from 0% to 30%, it decreased between 27.5%. However, reductions of up to 39.8% and 39.5% in the load-carrying capacity occurred respectively compared with RCBs with the longitudinal tension reinforcements of 2φ10 and 2φ12 without CP. The study concluded that more than 10% CP cannot be used without precautions for mixtures.

Qaidi et al. [23] discussed the usage of waste glass as a partial or complete replacement for aggregates in the production of concrete. They reviewed the literature regarding the use of recycled glass waste in concrete and the effects that it has on its fresh and mechanical properties. Their research was focused on the benefits of using waste glass in concrete construction and its potential to create sustainable buildings. They concluded that although adding waste glass to the concrete mixture can ameliorate certain mechanical properties of concrete, decreases concrete dead load, and acts as an ecological replacement for normal aggregates.

Celik et al. [24] investigated the effectiveness of replacing coarse and fine aggregates with ground glass powder and crushed waste glass, with proportions of 10%, 20%, 40%, and 50%. The mechanical properties were tested, including compression, splitting tensile, and flexural tests. Glass powder caused a better pozzolanic effect and increased the strength while glass particles decreased the strength when replaced with aggregates. With a 14% increase in the tensile strength, replacing fine aggregates with waste glass particles was found to be effective. The flexural strength increased by 3.2%, 6.3%, 11.1%, and 4.8% when 10%, 20%, 40%, and 50% of fine aggregates were replaced with waste glass. In addition, a number of voids were formed in the specimens where large glass pieces were replaced with aggregate, which negatively affected the strength. Based on the results, a 20% replacement for fine and coarse aggregates with waste glass is recommended.

Celik et al. [25] examined the impact of incorporating waste glass powder (WGP) with fly ash in various proportions on geopolymer concrete (GPC). They aimed to evaluate the effect of using different proportions of molarity and WGP percentages in GPC. For this purpose, they tested the workability, setting time, and splitting tensile and flexural strengths of GPC incorporating WGP and NaOH molarity. The findings revealed that the workability was reduced with increasing WGP percentages. It was also found that M13 NaOH with 10% WGP provided the optimum sustainable GPC in terms of both fresh and hardening properties. Furthermore, the study revealed that while NaOH molarity increased the compressive strength, it had a negative impact on the setting time and workability. SEM analysis was performed on the specimens, which confirmed the results.

Chang et al. [26] discussed the utilization of ceramic waste in partial cement replacement to reduce the consumption of natural resources and carbon emissions in cement production. The supervised machine learning algorithms such as Decision Tree, AdaBoost, Bagging, Random Forest, Gradient Boosting, and XGBoost were employed for predicting the Compressive Strength (CS) of ceramic waste powder concrete (CWPC) and the k-fold cross-validation technique was applied afterwards. The study found that the Random Forest algorithm was the most effective with a higher R^2^ value of 0.97 and significantly lesser RMSE and MAE values of 1.40 and 1.13, respectively. The study concluded that the concrete with 10% CWP content could have reduced impacts on natural resources, climate change, ecosystem quality, and human health, and the effect on non-renewable energy resources, depletion of the ozone layer, and global warming can be reduced by up to 7%, 6%, and 9%, respectively. The application of ML techniques in civil engineering can provide benefits in terms of conserving resources, effort, and time.

**Fig. 1:**
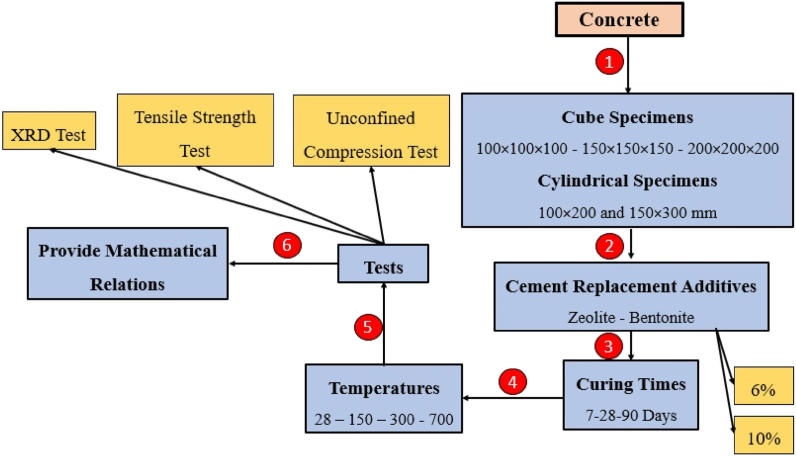
Representation of experimental procedures.

Based on the literature review, mechanical properties of the concrete specimens containing zeolite and bentonite under elevated temperatures, have not been investigated yet. On the other hand, although the ratios for converting compressive strength of the cube to the cylinder strength are available in the literature, such ratios have not been developed for the concretes containing pozzolans such as zeolite or bentonite or at expose to high temperatures. Consequently, the current paper investigates the effect of incorporating zeolite and bentonite on the behavior of cubic and cylindrical specimens under rising temperatures. Additionally, this study proposes a way to convert the compressive strength of cubic specimens to those of cylindrical ones containing pozzolans. Fig. 1 outlines the procedures adopted in this study.

## 2 Experimental program

In this study, four concrete design mixes with cement volume of 250kg/m3, were taken into account for the cubic and cylindrical specimens. One of the concrete mixes is the ordinary concrete and the rest of them, contain zeolite and bentonite. To produce the pozzolanic concrete, water-to-cement (W/C) ratio of 0.65 was used. Moreover, 6 and 10% cement were added with bentonite and zeolite (6 and 10% for each material). These add ratios have been considered based on the previous studies [27]. Furthermore, ratio of the fine to coarse grains in the concrete mixes, was set to 0.33 to 0.67. The compressive and tensile strength tests were conducted on the cubic and cylindrical specimens at the ages of 28 and 90 days. Size of the cubic specimens was considered equal to 100 × 100 × 100, 150 × 150 × 150 and 200 × 200 × 200 mm. Moreover, the cylindrical specimens were built in sizes of 200 × 100 and 300 × 150 mm. All specimens were exposed to temperatures of 28, 150, 300 and 700 °C (The temperatures were selected based on the study of past research and related to the topic of this paper.).

### 2.1 Material properties

**Fig. 2:**
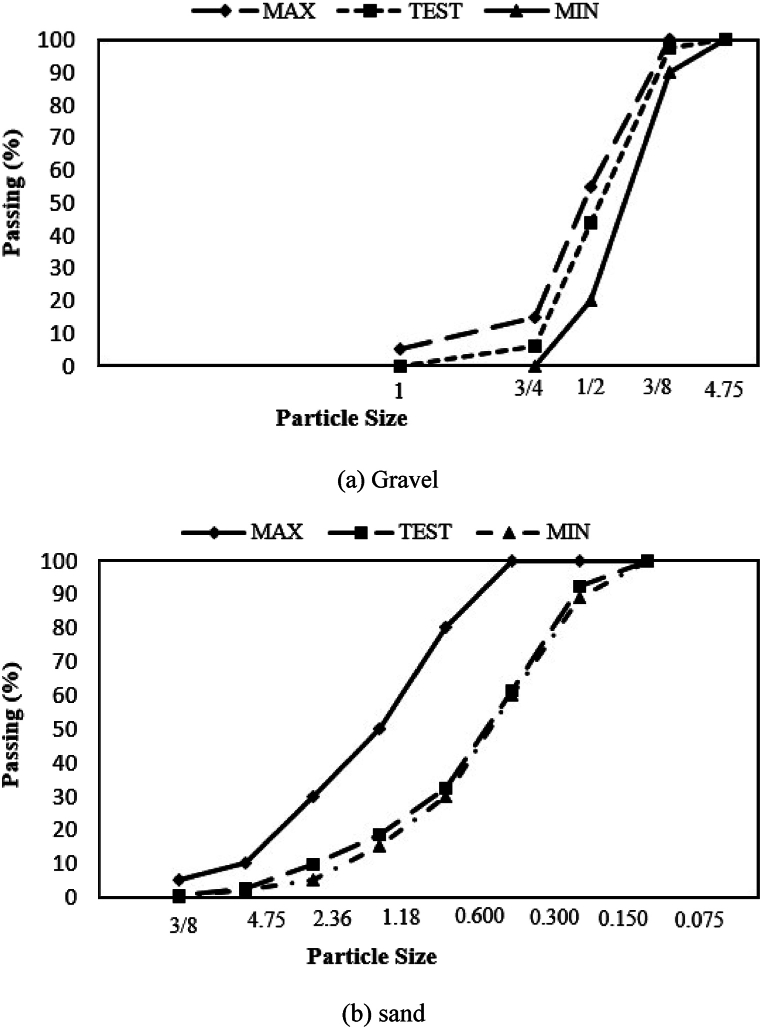
Particle size distribution curve.

To specify grading of the aggregates, respective tests have been performed according to ASTM-C33 [28] and the particle size distribution curve is presented in Fig. 2. The maximum nominal size of the gravel ranges between 8 and 9 mm (Fig. 2a and b).

Cement type II was used to produce the mortar. Moreover, color of the zeolite used in the concrete mixes, is cream, whose density is equal to 2.3gr/cm3. Similarly, color of the sodium-incorporated bentonite is cream, which has been prepared from mines of Semnan city. Chemical composition of the zeolite, bentonite and cement is given in Table 1.

**Table 1:** Chemical composition of cement, zeolite and bentonite (%).

Compounds	Cement	Zeolite	Bentonite
SiO_2_	21.11	69.28	69.8
Al_2_O_3_	4.42	10.43	11.88
Fe_2_O_3_	3.96	0.49	1.73
CaO	63.23	3.56	0.96
MgO	1.51	0.5	1.42
Na_2_O	0.32	0.73	0.5
K_2_O	0.51	1.27	0.47
TiO_2_	–	0.166	0.1

**Fig. 3:**
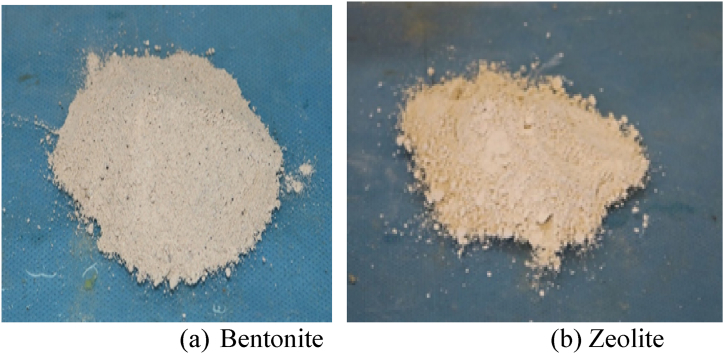
Substitute materials for cement.

The zeolite and bentonite that have been used as add for cement, are demonstrated in Fig. 3a and b.

## 3 Concrete design mix ratios and construction process

To evaluate effect of heat on the concrete properties, the specimens were prepared using one mix design and cured under the same conditions. Accordingly, the mix designs are given in Table 2 That the basic mixing plan is chosen based on the mixing plan of common projects in Iran.

**Table 2:** Concrete mix design (kg/m^3^).

	Specimen	Sand	Gravel	Cement	Water	Bentonite	Zeolite
0.65: Water to cement ratio	0.65-C250	1240	620	250	162.5	0	0
	0.65–6b6z	1240	620	250	162.5	15	15
C250: cement grade 6b: 6% bentonite	0.65–10b6z	1240	620	250	162.5	25	15
6z: 6% zeolite 10b: 10% bentonite 10z: 10% zeolite	0.65–6b10z	1240	620	250	162.5	15	25

The specimens have been prepared according to ACI211. In addition, ratio of water-to-cementitious materials is et to 0.65 which is in compliance with the results of numerous tests and characteristics of the materials such as zeolite and bentonite.

In order to mix the materials, first dry sand and gravel were mixed for 1 min. Then, one-third of the mix design's water was added to build a uniform mixture and afterwards, dry cement and pozzolans that had been already mixed, were added to the mixer. Next, two-third of the remaining water was slowly added and all materials were mixed for 2 min.

After preparation of the specimens, slump test was conducted on the fresh concrete according to ASTM C143 [29]. Based on the results, slump value varies between 6 and 8 cm. To build the specimens, the formworks were first lubricated, enabling the concrete mixes to be easily demoulded. In order to compact the concrete, the mixture was poured into the formwork in three layers and each layer was impacted. All specimens remained in the laboratory for 24 h and after demoulding, the concrete mixes were immersed into water to be cured for ages of 7, 28 and 90 days [30].

**Fig. 4:**
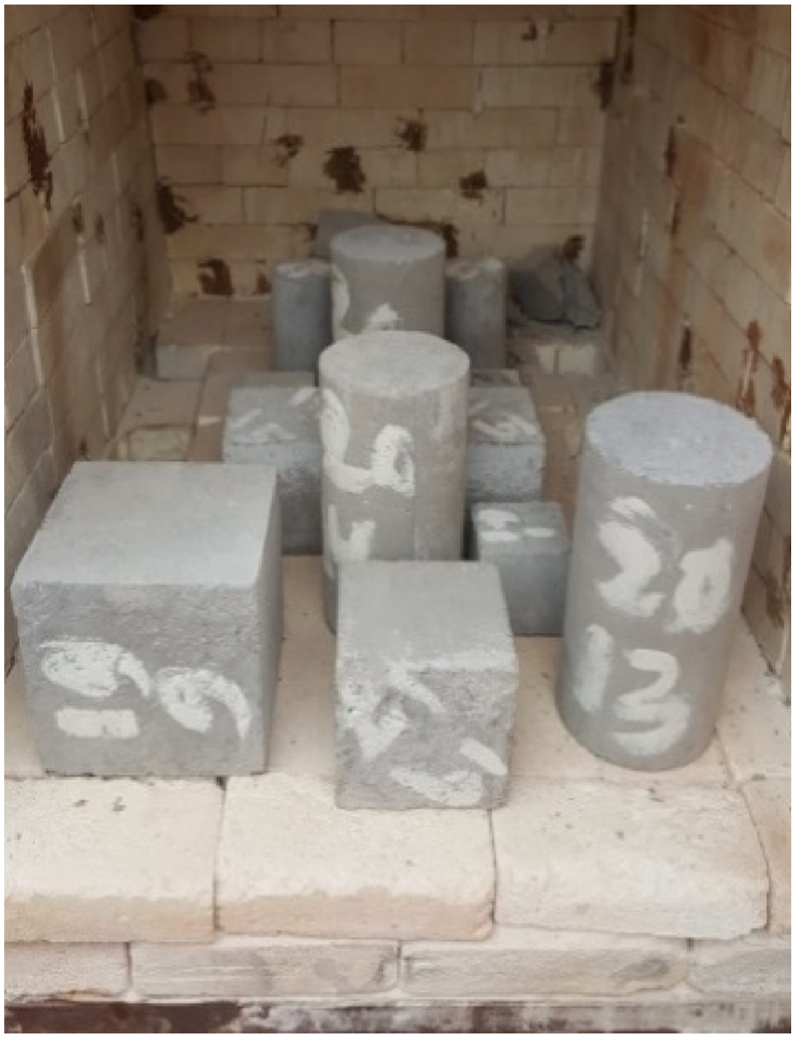
Specimens inside the furnace.

Compressive strength test was carried out on the cubic and cylindrical specimens at the ages of 7, 28 and 90 days and subsequently, the tensile strength test was conducted on the cylindrical specimens at the ages of 28 and 90 days. To study the effect of heat on the concrete mixes, all specimens were exposed to temperatures of 28, 150, 300 and 700 °C. In Fig. 4, photos of the specimens inside the electric furnace are shown.

The target temperatures have been provided using an electric furnace with thermal capacity of 1300 °C. Accordingly, the time required that both temperatures, inside the furnace and concrete core, reach the temperature of interest, is nearly 1 h. For this purpose, when the temperature inside the furnace reaches the temperature of interest, the specimens remained at that temperature for 1 h so that temperatures of the inner and outer parts of the specimens become the same [31,32].

This procedure is adopted to get the specimens slowly cooled off. As a result, the furnace was shut down for 1 h and then, the specimens were gradually taken out. Importantly, occurrence of thermal shock is avoided in this way. After that, the specimens remained in the ambient temperature for 24 h and then, the tests were conducted on them.

## 4 Specifications of the tests

**Fig. 5:**
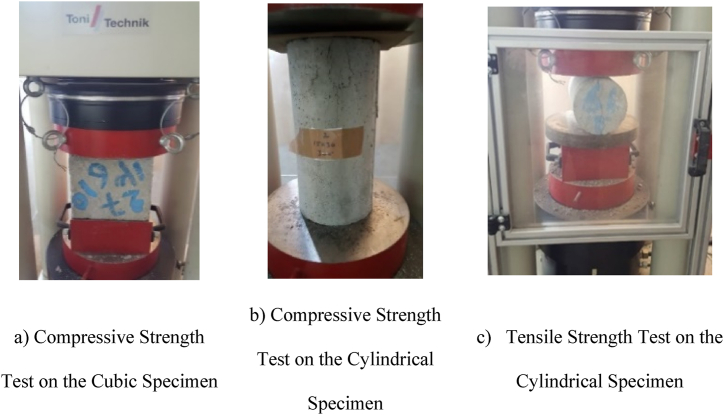
Specimens during the strength tests.

In this study, the compressive strength tests were carried out on the cubic specimens with size of 100 × 100 × 100, 150 × 150 × 150 and 200 × 200 × 200 mm, in accordance with BS12390-3 [33]. Moreover, based on ASTM C39, compressive strength tests were conducted on the cylindrical specimens with size of 200 × 100 and 300 × 150 mm [34]. However, tensile strength tests on the cylindrical specimens with size of 300 × 150 mm were performed conforming to ASTM C496 [35]. The specimens during the strength tests are illustrated in Fig. 5a–c.

## 5 Results

### 5.1 Compressive strength

#### 5.1.1 Cubic specimens

**Fig. 6:**
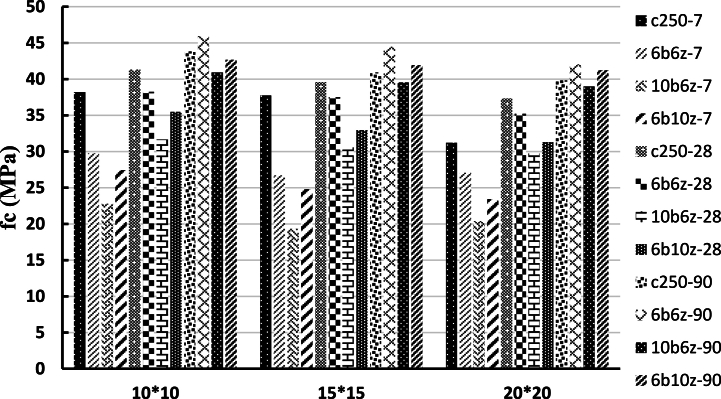
Compressive Strength of Cubic Specimens at the ages of 7, 28 and 90 days under ambient temperature.

Results of the average compressive strength of the cubic specimens at the ages of 7, 28 and 90 days under ambient temperature, for the four mix designs of C250, 6b6z, 10b6z and 6b10z have been presented in Fig. 6. As can be seen in Fig. 6, the compressive strength of all cubic specimens containing pozzolans has decreased compared to that of the control specimen. This behavioral trend is completely normal because chemical reaction of the pozzolans takes a longer time in comparison with the control specimen and at the age of 7 days, the pozzolans-incorporated specimens could not be expected to attain an excellent strength (The property of added pozzolanic materials is such that it takes more time to perform hydration and pozzolanic reactions than cement, so at the age of 7 days, we cannot expect the same performance as cement from them. Therefore, at older ages, by performing the necessary reactions, it will reach the desired resistance).

At the age of 28 days, among the cubic specimens, compressive strength of 6b6z and 6b10z is about 90 and 80% of the strength of the control specimen and also, the minimum compressive strength at the age of 28 days, has occurred for 10b6z.

At the age of 90 days, it was observed that 6b6z and 6b10z have experienced 5% increase in their strength compared to the control specimen but strength of 10b6z was equal to 90% of the strength of the control specimen. Study of the results indicate that incorporation of pozzolans reduce the strength and at the older ages, the difference in the compressive strength decreases or in some cases, the strength increases compared to the control specimen.

**Fig. 7:**
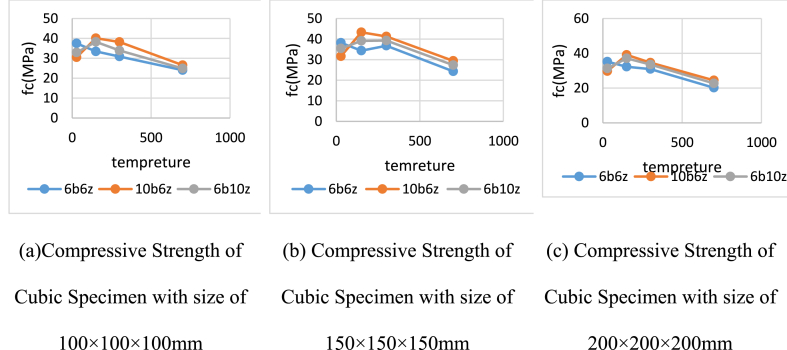
Compressive Strengths of Cubic Specimens under 28, 150, 300 and 700 °C at the age of 28 days.

Comparing the compressive strength of the cubic specimens indicate that the specimen with size of 100 × 100 × 100 mm has the maximum strength and the specimen with size of 200 × 200 × 200 has the minimum value. According to the stress equation which is the force divided by the cross-sectional area, it can be stated that the smaller the area is, the greater the stress becomes, which confirms accuracy of the findings herein. Results of the compressive strength of the cubic specimens under 28, 150, 300 and 700 °C at the age of 28 days, are presented in Fig. 7a–c.

It can be observed that the specimen containing 10% bentonite has the maximum compressive strength under 150, 300 and 700 °C. Rate of increase in the compressive strength of all cubic specimens at 150 and 300 °C compared to the unheated 10b6z at the age of 28 days, is equal to 23 and 24%, respectively. However, under 700 °C, in comparison with the unheated 10b6z at the age of 28 days, the strength has decreased by 13%.

In the case of 6b10z, it can be observed that 28-day strength of the heated specimen under 150 and 300 °C, has increased by 16% compared to the unheated ones but under 700 °C, its strength decreases by 30%. Accordingly, strength reduction at 700 °C, could be attributed to evaporation of water existing in the specimens. Heating 6b6z specimen at the age of 28 days, has reduced the strength at all temperatures of 150, 300 and 700 °C. Results of compressive strength of the cubic specimens under temperatures of 150, 300 and 700 °C at the age of 90 days, are presented in Table 3.

**Table 3:** Compressive Strengths of Cubic Specimens under 300 and 700 °C at the age of 90 days.

	Cube100x100x100 (mm)		Cube 150x150x150 (mm)	
Specimen	Fc (MPa) 300	Fc (MPa) 700	Fc (MPa) 300	Fc (MPa) 700
C250	42.80	35.76	41.71	32.56
6b6z	39.32	30.52	37.82	27.37
6b10z	37.52	28.51	35.41	23.8
10b6z	34.64	25.19	33.44	20.26

As can be observed, at the age of 90 days, strength of all heated cubic specimens decreased compared to the unheated ones. Based on the results, under 300 °C, the control specimen has the maximum compressive strength and 10b6z is of the lowest strength.

Strength of the cubic specimens containing C250 under 300 °C, has decreased by 3% compared to the control specimen (Due to the non-activation of the pozzolanic reactions of the added pozzolans). Cubic specimens 6b6z, 10b6z and 6b10z under 300 °C, have experienced a reduction by 15% at the 90-day strength in comparison with the unheated specimens.

Similar to the trend observed for temperature of 300 °C, the compressive strength decreases under 700 °C but rate of reduction is greater. Under exposure to 700 °C, compressive strength of the control specimen, 6b6z, 10b6z and 6b10z has dropped by 19, 36, 44 and 38%, respectively.

In general, the results indicate that temperature of 700 °C at the age of 90 days, significantly reduces compressive strength of the pozzolan-incorporated specimens but the control specimen is not remarkably affected by the heat. Moreover, it can be seen that 28-day strength of 6b6z has increased compared to the age of 90 days but the other specimens have experienced an opposite behavioral trend.

Generally, it can be seen that exposed to 150 and 300 °C, all specimens have gained a better performance. In this respect, at the age of 28 days, strength of 10b6z and 6b10z has increased under 150 °C and the results are quite close to those of the specimens tested under exposure to 300 °C. However, exposure to temperature of 700 °C, has led to decrease in strength of all specimens. Evaluation of the results reveal that if specimens are heated and thus, their strength is reduced, then, incorporation of zeolite and bentonite could compensate for this reduction. In this respect, inclusion of these two materials manages to increase the 28-day strength of the concrete specimens.

In the codes accounting for effect of heat on mechanical properties of the concrete, compressive strength of ordinary concrete has been considered as the normalized strength. The normalized strength is equal to the strength at any temperature divided by the strength at ambient temperature. In these codes, the values are based on the tests carried out on the cooled specimens. The normalized compressive strength of the tested specimens and those presented in En1992 and AISC360-10 are presented in Table 4 [35]. In the table, the symbol H represents the heated specimens and the symbol R indicates the corresponding heated specimens.

**Table 4:** Normalized Compressive Strength of Cubic Specimens and the values presented by the Codes.

					Cu 100x100x100 (mm)	Cu 150x150x150 (mm)	Cu 200x200x200 (mm)
SI·NO.	Specimens	temperature	En1992-1-2	AISC 360-10	$\frac{\text{fc},28\left(H\right)}{\text{fc},28\left(R\right)}$	$\frac{\text{fc},28\left(H\right)}{\text{fc},28\left(R\right)}$	$\frac{\text{fc},28\left(H\right)}{\text{fc},28\left(R\right)}$
1	6b6z	150	0.98	0.93	0.89	0.89	0.92
2	6b10z	150	0.98	0.93	1.1	1.16	1.22
3	10b6z	150	0.98	0.93	1.37	1.31	1.31
4	6b6z	300	0.91	0.84	0.96	0.82	0.83
5	6b10z	300	0.91	0.84	1.1	1.03	1.07
6	10b6z	300	0.91	0.84	1.3	1.25	1.16
7	6b6z	700	0.43	0.3	0.64	0.64	0.57
8	6b10z	700	0.43	0.3	0.77	0.74	0.73
9	10b6z	700	0.43	0.3	0.93	0.87	0.82

In all cubic specimens with size of 100 × 100 × 100 mm except for 6b6z, 28-day normalized compressive strength has increased under exposure to 150 °C.

In the case of 200 × 200 × 200 specimens, it can be observed that normalized strength of 6b6z at 150 and 300 °C, is less than the value proposed by En1992-1-2 but the results are quite the same with those derived from AISC 360-10. Besides, under exposure to 700 °C, the normalized compressive strength has increased in comparison with the values obtained from both codes.

The normalized compressive strength of 6b10z and 10b6z has increased under exposure to all temperatures compared to the values proposed by both codes. In these specimens, similar to the cubic specimens with size of 100 and 150 mm, incorporation of 10% bentonite significantly increases compressive strength of the heated specimens which indicates the positive consequences of replacing cement with zeolite and bentonite.

**Fig. 8:**
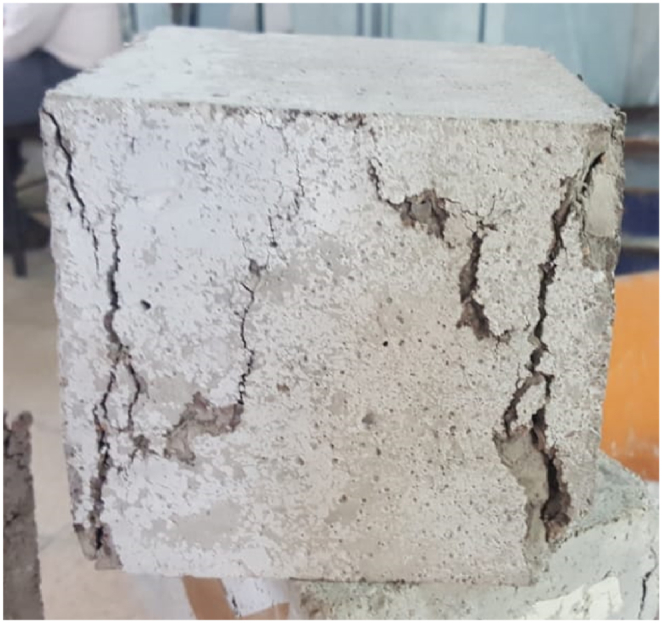
How to break some cubic specimens.

The manner of failure and also the flaking of some specimens after being get out from the electric furnace and performing the compressive strength test are presented in Fig. 8.

#### 5.1.2 Cylindrical specimens

**Fig. 9:**
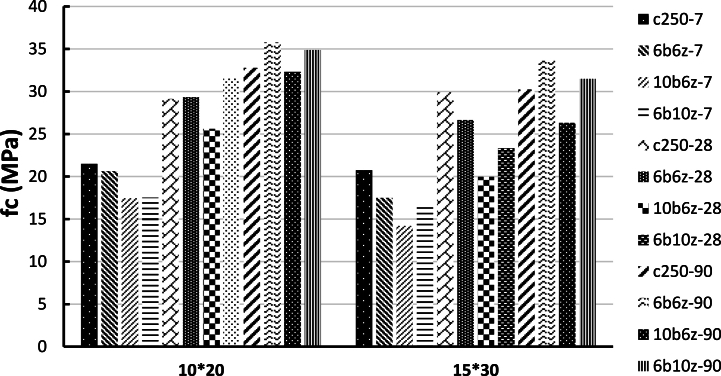
Compressive Strength of Cylindrical Specimens at the ages of 7, 28 and 90 days under ambient temperature.

The average compressive strength of the cylindrical specimens at the ages of 7, 28 and 90 days under ambient temperature and for four concrete mixes named as C250, 6b6z, 10b6z and 6b10z are shown in Fig. 9. Similar to the cubic specimens, 7-day strength of 6b6z, 6b10z and 10b6z in both sizes (i.e. 200 × 100 and 300 × 150 mm) has dropped compared to the control specimen. However, 6b6z has gained greater strength and reached 90% of the control specimen's strength.

The specimen containing 10% bentonite (10b6z) has gained a lower strength at the age of 7 days compared to the other two concrete mixes. At the age of 28 days, the 200 × 100 mm specimens containing 6 and 10% pozzolans (i.e. 6b6z and 6b10z) have the same strength in comparison with the control specimen (C250). Notably, the lowest strength at the age of 28 days, belongs to 10b6z.

In the case of cylindrical specimens with size of 300 × 150 mm, 28-day strength of 6b6z and 6b10z is equal to 89 and 75% of strength of the C250, respectively and lowest strength has occurred for 10b6z. At the age of 90 days, it is observed that strength of 6b6z and 6b10z has increased by 9% and strength of 10b6z is approximately equal to 90% of strength of the control specimen. This finding indicates that the pozzolanic activity of bentonite takes longer than that of zeolite and as the specimens becomes further aged, difference between the strengths is reduced or in some cases, increases the strength.

**Fig. 10:**
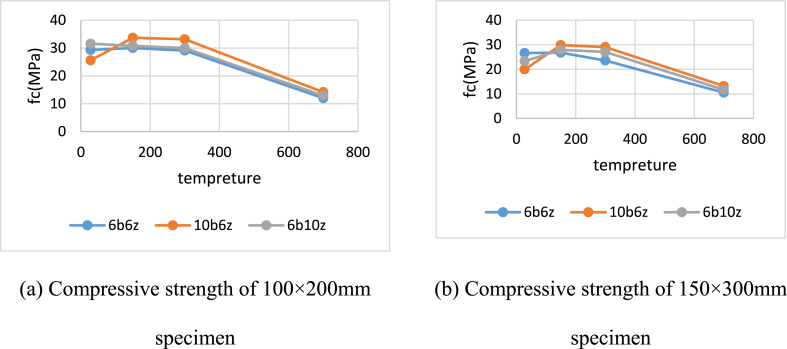
28-day Compressive Strength of Cylindrical Specimens under exposure to 28, 150, 300 and 700 °C.

As mentioned earlier, according to the stress equation, the smaller cross-sectional area leads to greater rates of stress. For instance, the cubic specimens have smaller rates of strength compared to the cylindrical ones. The compressive strengths of the cylindrical specimens under exposure to 28, 150, 300 and 700 °C at the age of 28 days, are presented in Fig. 10a and b.

In the case of the cylindrical specimen with diameter of 100 mm, the specimen containing 10% bentonite has the highest strength under exposure to all temperatures of 150, 300 and 700 °C. Moreover, 28-day strength of this specimen under exposure to 150 and 300 °C has increased by 31 and 29%, respectively, compared to that of the unheated specimen. The value of this increase for the specimen with diameter of 300 mm, is equal to 46% approximately.

In the case of the cylindrical specimens with both sizes, the strength of the specimen containing 10% bentonite at 700 °C, has decreased up to 45% in comparison with 10b6z. similarly, strength of 6b10z under 150 and 300 °C, has increased by 5–10% compared to the unheated cases. In the case of the specimens containing 6% bentonite and zeolite (6b6z), under 150 and 300 °C, better results have been obtained compared to the cubic specimens. Based on the results, the compressive strength at these temperatures is almost the same as that of the control specimen but at 700 °C, the strength has dropped by 58% compared to the control specimen. The compressive strengths of the cylindrical specimens exposed to 150, 300 and 700 °C at the age of 90 days, are presented in Table 5.

**Table 5:** Compressive Strength of Cylindrical Specimens under exposure to 300 and 700 °C.

	Cylinder 100x200 (mm)	
Name	Fc (MPa) 300	Fc (MPa) 700
C250	31.5	25.18
6b6z	32.1	21.74
6b10z	27.93	18.32
10b6z	25.9	14.79

In Table 6, values of the normalized compressive strength of the cylindrical specimens as well as those obtained from En 1992 and AISC 360-10 are given.

**Table 6:** Normalized Compressive Strength of the Cylindrical Specimens and the values obtained from the Codes.

					Cy 100x200 (mm)	Cy 150x300 (mm)
SI·NO.	samples	temperature	En1992-1-2	AISC 360-10	$\frac{\text{fc},28\left(H\right)}{\text{fc},28\left(R\right)}$	$\frac{\text{fc},28\left(H\right)}{\text{fc},28\left(R\right)}$
1	6b6z	150	0.98	0.93	1.02	1
2	6b10z	150	0.98	0.93	0.97	1.20
3	10b6z	150	0.98	0.93	1.32	1.49
4	6b6z	300	0.91	0.84	0.99	0.89
5	6b10z	300	0.91	0.84	0.95	1.16
6	10b6z	300	0.91	0.84	1.29	1.45
7	6b6z	700	0.43	0.3	0.43	0.42
8	6b10z	700	0.43	0.3	0.41	0.50
9	10b6z	700	0.43	0.3	0.55	0.60

According to the results, the values of 28-day normalized strength of all specimens under all

temperatures have either increased based on both codes or approximately, the same result has been achieved. In this section, it can be observed that accuracy of using the pozzolans, can be approved.

**Fig. 11:**
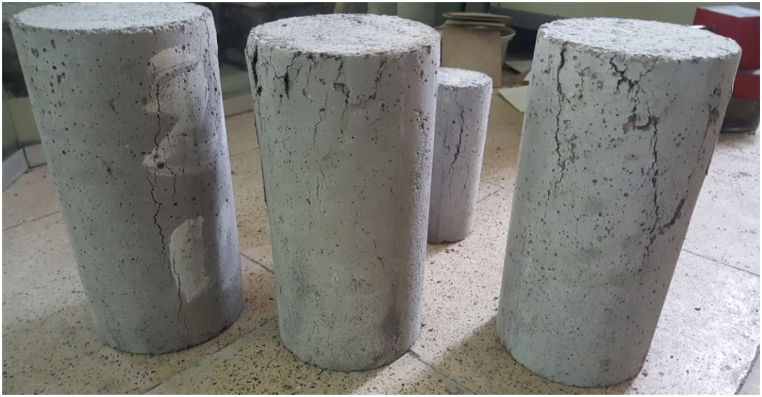
How to break some cylindrical specimens.

The manner of failure and also the flaking of some cylindrical specimens after being get out from the electric furnace and performing the compressive strength test are presented in Fig. 11.

#### 5.1.3 Study of compressive strength of cubic and cylindrical specimens in terms of their size

**Fig. 12:**
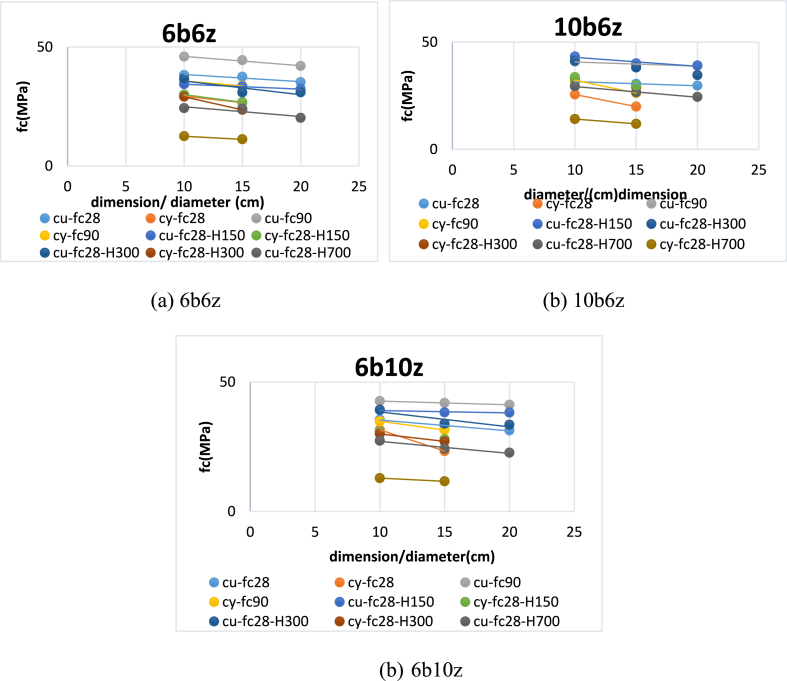
Variations of Cubic and Cylindrical Specimens' Strength based on Change in Size of Specimens.

In Fig. 12, regression analysis of the data for 6b6z, 6b10z and 10b6z has been presented. It can be seen that as the strength increases, slope of the regression line is reduced. It means that rate of reduction in strength has decreased following increase in size of the specimen and the regression line tends to a direct line. To justify the greater strength of the cubic specimens, it can be stated that due to friction between the loading steel plate and the loaded area as well as difference between properties of these two materials, shear stresses are developed at the contact area of the specimen with the plates. It has been already proved that the shear stress grows with maximum angle of 60°. In the cubic specimens, the shear stresses are developed from top and bottom of the specimen and meet each other in the middle of the specimen's height. However, in the cylindrical specimens, there is an area in the middle on which, there are no signs of shear stresses. This fact makes the cylindrical specimens offer lower strength. Moreover, based on the probability theory, as size of the specimen increases, generation of cracks becomes more likely. Despite the fact that the concrete is under compression, its failure is due to tension and consequently, as generation of cracks becomes more likely, occurrence of failure at lower strengths, becomes more probable.

### 5.2 Tensile strength test

**Fig. 13:**
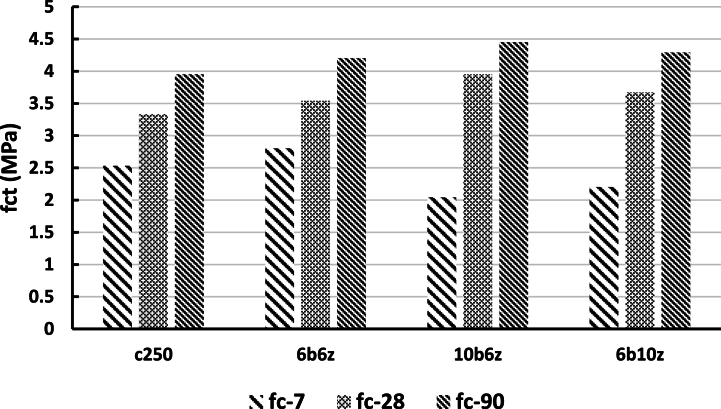
Tensile Strengths under Ambient Temperature at the ages of 7, 28 and 90 days.

The tensile strength tests were carried out based on ASTM C496. Accordingly, the results for the 300 × 150 mm specimens at ambient temperature and for ages of 7, 28 and 90 days are presented in Fig. 13. The formula used to calculate the tensile strength is as follows (eq. (1)):

(1) $$f_{t}=\frac{2p}{\pi .L.D}$$

Where ft, P, L and D represent the tensile strength (MPa), maximum applied load (N), length and diameter (mm) of the cylindrical specimen.

Based on the results, it can be observed that at the age of 7 days, tensile strength of 6b6z has increased compared to the control specimen, and has the maximum strength. Furthermore, it can be seen that 10b6z and 6b10z have a lower strength in contrast to the control specimen. Comparing the 28-day strengths indicate that the pozzolanic materials substantially affect the materials adhesion. Similarly, at the age of 28 days, the specimen containing 10% bentonite has a higher strength compared to the other specimens and quantitatively, its strength has increased by 20% in comparison with the control specimen. At the age of 28 days, it is observed that the control specimen (C250) has the lowest strength. The strength values of 6b6z and 6b10z are greater than that of the control specimen by 6 and 10%, respectively. Notably, plasticity of bentonite is greater than that of zeolite which has led to formation of a flexible layer around the aggregates and thus, deformability of the specimens has improved. The trend of increase in strength of the specimens at the age of 90 days, is similar to that the age of 28 days; in such a way that strength of 10b6z, 6b10z and 6b6z has increased up to 11, 6 and 4% compared to C250. The tensile strengths of the pozzolans-incorporated specimens under exposure to 150, 300 and 700 °C at the age of 28 days, are given in Table 7.

**Table 7:** 28-Day Tensile Strength Values under Exposure to various temperatures.

Name of Sample	Fct (MPa) 150	Fct (MPa) 300	Fct (MPa) 700
6b6z	2.94	2.6	0.72
6b10z	3.7	3.25	0.83
10b6z	4.0	3.64	0.83

Similar to the compressive strength, the tensile strength of the specimen containing 10% bentonite at 150, 300 and 700 °C, have the maximum values. Tensile strength of 10b6z under 150 °C, has increased by 2% compared to the unheated specimen but, at the 300 °C, the strength has dropped by 8%. Rate of strength reduction under exposure to 700 °C, is significant for this specimen as it has reached the value of 0.87. similar to the specimen containing 10% bentonite, tensile strength of 6b10z at 150 °C, is approximately the same as strength of the unheated specimen and it has increased only 2% but under 300 °C, the strength has decreased up to 13%. Temperature of 700 °C has remarkably reduced tensile strength of 6b10z. Exposure to temperatures of 150 and 300 °C, has decreased the strength of 6b6z by 20 and 36%, respectively. In general, the specimens containing 10% bentonite and zeolite at 150 °C, have similar results in comparison with the unheated specimens and under exposure to 300 °C, the strength is not severely reduced. On the contrary, when temperature rises to 700 °C, the strength decreases significantly. The values of the normalized tensile strength of the 300 × 150 mm specimens and those obtained from En 1992 are given in Table 8.

**Table 8:** Normalized Compressive Strength of 300 × 150 mm Specimens and the values obtained from the Codes.

SI·NO.	samples	temperature	En1992-1-2	$\frac{\text{fc}t,28\left(H\right)}{\text{fct},28\left(R\right)}$
1	6b6z	150	0.99	0.83
2	6b10z	150	0.99	1.01
3	10b6z	150	0.99	1.01
4	6b6z	300	0.6	0.83
5	6b10z	300	0.6	1.01
6	10b6z	300	0.6	1.01
7	6b6z	700	0	0.2
8	6b10z	700	0	0.22
9	10b6z	700	0	0.23

### 5.3 Proposed relationships to determine the compressive and tensile strengths based on temperature

As the values obtained for the compressive and tensile strengths are properly consistent, through regression analysis of the results at any temperature, has led to development of relationships to determine the compressive and tensile strength of the pozzolanic concrete specimens as given in Table 9.

**Table 9:** The Relationships proposed to determine the Compressive and Tensile Strengths based on Temperature.

Specimen's Name		Regression Function	Correlation Coefficient (R^2^)
Compressive Strength	6b6z	y = −0.03x + 31.59	0.99
	6b10z	y = −0.03x + 34.28	0.95
	10b6z	y = −0.03x + 36.91	0.95
Tensile Strength	6b6z	y = −0.004x + 3.69	0.98
	6b10z	y = −0.005x + 4.66	0.98
	10b6z	y = −0.006x + 5.12	0.97

With respect to similarity of the relationships for the mentioned three mix designs, the regression function for estimating the compressive strength based on temperature is as follows (eq. 2):

(2) $$F_{C}=-0.0317T+34.25\;R^{2}=0.93$$

Moreover, the regression function to specify the tensile strength is as follows (eq. (3)):

(3) $$F_{T}=-0.0052T+4.4872\;R^{2}=0.9$$

### 5.4 Ratios for conversion of cube to cylinder strengths

**Fig. 14:**
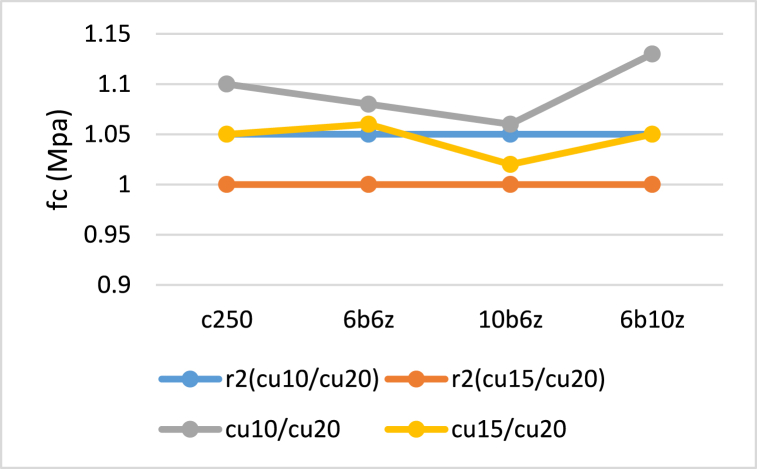
Comparison between Experimental and Code-based r2 values.

**Fig. 15:**
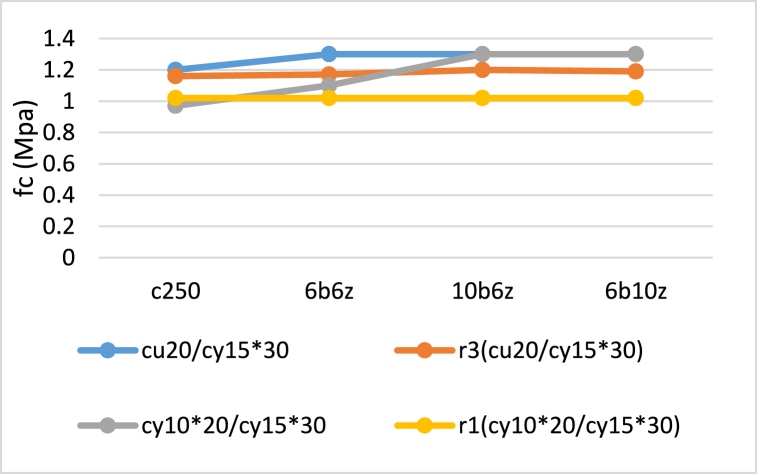
Comparison between Experimental and Code-based r1 and r3 values.

Fig. 14, Fig. 15 compare the r1, r2 and r3 factors obtained from the tests and Iranian concrete code which is significantly in consistency with ACI 318-19. It can be observed that difference rate of r2 and r3 in the specimens, is less than 10 and 8%, respectively. Moreover, it can be seen that difference rate of r1 in 6b6z and C250 is less than but this rate for 6b10z and 10b6z is roughly 20%.

#### 5.4.1 X-Ray Diffraction (XRD) analysis

**Fig. 16:**
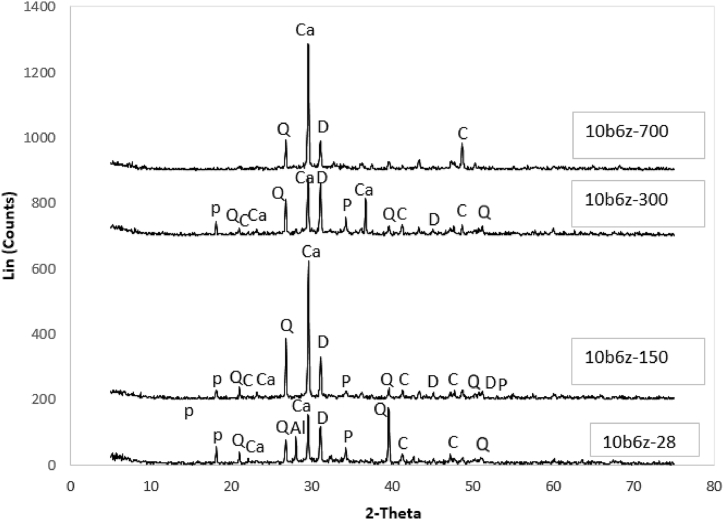
XRD Analysis Results for 10b6z under various temperatures.

Results of the XRD test on 10b6zunder exposure to temperatures of 28–700 °C, are presented in Fig. 16.

Comparing the results acquired from the XRD test results for the control specimen and the specimens containing bentonite and zeolite, it is observed that inclusion of these two materials has led to pozzolanic reactions among Al_2_O_3_, SiO_2_ and Ca(OH)_2_.

When Ca (OH)_2_ is consumed, its content is reduced and instead, amount of C–S–H gel increases. When the mortar is subjected to high temperatures, chemical variations occur and accordingly, its microstructure is detrimentally affected which finally leads to the structure's collapse. The XRD patterns detect mechanical properties of the phases such as Portlandite, C–S–H gel, Ca(OH)_2_, SiO_2_, CaMg(CO₃)₂ and NaAlSi_3_O_8_ – CaAl_2_Si_2_O_8_.

Mechanical properties of the cement paste is highly affected by the chemical bond of C–S–H gel. Evaluation of C–S–H peaks could help to better interpret the findings. In the case of 10b6z under ambient temperature, NaAlSi_3_O_8_– CaAl_2_Si_2_O_8_is observed but when temperature rises, this material transforms and turns into Ca (OH)_2_, whose inclusion has enhanced the compressive strength. Peak of SiO2 in the XRD pattern, is formed due to breakage of the components.

Peak intensity of C–S–H has dropped as per rise in temperature from 28 to 700 °C. These values reveal that peak intensity of C–S–H up to 300 °C, is approximately the same but under 700 °C, it has reduced considerably. Chemical variations in the microstructure under temperature lower than 300 °C, are insignificant. As a result, the strength loss at this temperature might arise from excessive evaporation of water, causing severe porosity in the concrete. The XRD patterns at 150 and 300 °C indicate a decrease and an increase in the content of Portlandite, respectively. Moreover, the intensification of Portlandite at 300 °C suggests that this material's low water reaction is reversible. This phase may be rectified within the timeframe that the specimens are being cooled down.

## 6 Comparison with previous research

To better understand the results obtained from the findings of this study, the best possible case in the results of this study are compared with the best possible case in the results of other researchers in Table 10. All issues mentioned in the Table are in percentage and are presented in comparison with the reference specimen related to the same paper. Notably, all research mentioned in the Table has used the same additives. Accordingly, the importance of the results obtained in this paper could be more comprehensively understood. In almost all cases, the results obtained from this paper are better than other studies and have improved the properties of the mortar several times more.

**Table 10:** Comparison of the results obtained in this paper with those of other research (%).

	Details	Compressive Strength		Tensile Strength	
		Bentonite	Zeolite	Bentonite	Zeolite
This Paper	Concrete	+63.6	+63.6	+110	+110
[9]	Concrete	+20	–	+22.7	–
[10]	Concrete	+4.1	–	−11.8	–
[15]	Concrete	–	+57.7	–	–
[18]	High-Performance Cement-Based Concrete	–	+22.7	–	–
[27]	Concrete	−8	−8	+10	+10

## 7 Conclusion

This paper investigated the effects of partially replacing cement with zeolite and bentonite in the concrete (four mix designs were studied) under elevated temperatures. Zeolite and bentonite were added to the cubic and cylindrical specimens at replacement ratios of 6 and 10%. After curing the specimens at the ages of 7, 28 and 90 days, they were exposed to the elevated temperatures (150, 300 and 700 °C), to carry out the mechanical properties tests and microstructure analysis. Moreover, the coefficients to convert the compressive strength of the cubic specimens to that of the cylindrical ones were recommended. The most important conclusions are as follows:

• The specimens containing 10% bentonite had the highest compressive strength at 28 days, subjected to the temperatures of 150, 300 and 700 °C. This led to a 25–36% increase in compressive strength for cubic and cylindrical specimens and a 29–48% increase for specimens exposed to 150 and 300 °C, respectively. Heat treating enhanced the compressive strength of specimens containing zeolite and bentonite by 10%.
• Tensile strength decreased at 90-day age, with exposure to 300 °C and 700 °C. Specimens at 300 °C had the highest compressive strength, but the specimen containing 10% bentonite exhibited the lowest. Compressive strength reduced between 30 and 40% at 700 °C compared to 300 °C.
• The experimental normalized compressive strength of the cubic and cylindrical specimens exposed to 150 and 300 °C, with 10% zeolite and bentonite, increased by 10–40%. This result illustrates the positive effect of using zeolite and bentonite on the normalized tensile strength.
• The tensile strength of specimens with 10% zeolite was highest at 150, 300, and 700 °C. Exposure to 150 °C increased strength of specimens containing 10% bentonite and 6% zeolite by 2%, but was reduced by 8% at 300 °C. Rate of decrease in tensile strength at 700 °C for specimens with 10% bentonite and zeolite was nearly 80%.
• In most cases, the difference between the scale factor of the cubic and cylindrical specimens is less than 10% compared to the value recommended by the standard.

## Compliance with ethical standards

This paper does not contain any studies with human participants performed by any of the authors.

## Funding

This study was not funded.

## CRediT authorship contribution statement

**Ghasem Pachideh:** Investigation. **Majid Gholhaki:** Resources. **Ahlam Aljenabi:** Methodology. **Omid Rezaifar:** Validation.

## Declaration of competing interest

The authors declare the following financial interests/personal relationships which may be considered as potential competing interests: Majidgholhaki reports financial support was provided by 10.13039/501100007103Semnan University. Majid gholhaki reports a relationship with Semnan University Faculty of Civil Engineering that includes: employment. If there are other authors, they declare that they have no known competing financial interests or personal relationships that could have appeared to influence the work reported in this paper.
